# Fetuses exposed to an episode of threatened preterm labor delivered at or near term show signs of cardiac remodeling

**DOI:** 10.1002/uog.70304

**Published:** 2026-08-16

**Authors:** C. Murillo, M. Palacio, C. Rueda, M. Larroya, D. Boada, J. Ponce, M. Guilleumes, O. Gómez, S. Ferrero, E. Gratacós, F. Crispi, T. Cobo

**Affiliations:** ^1^ BCNatal ‐ Barcelona Center for Maternal‐Fetal and Neonatal Medicine (Hospital Clinic and Hospital Sant Joan de Déu), Institut Clínic de Ginecología, Obstetrícia I Neonatología, Fetal i+D Fetal Medicine Research Center Barcelona Spain; ^2^ Fundació de Recerca Clínica Barcelona ‐ Institut d'Investigacions Biomèdiques August Pi I Sunyer (IIS‐FRCB‐IDIBAPS), Universitat de Barcelona Barcelona Spain; ^3^ Center for Biomedical Research on Rare Diseases (CIBER‐ER), Instituto Instituto de Salud Carlos III (ISCIII) Madrid Spain; ^4^ Department of Obstetrics, Gynecology, and Reproductive Medicine Hospital Universitari Dexeus Barcelona Spain

**Keywords:** amniotic fluid, echocardiography, fetal heart, infection, inflammation, natriuretic peptide, premature birth, troponin I

## Abstract

**Objective:**

To evaluate whether fetuses exposed to threatened preterm labor (PTL) without intra‐amniotic infection or inflammation (IAI), who were born ≥ 34 weeks' gestation, show signs of cardiac remodeling and dysfunction at admission and 4 weeks after the episode of threatened PTL.

**Methods:**

This was a prospective cohort study conducted at two tertiary referral centers including pregnant patients admitted between September 2018 and April 2021. The study group comprised women with singleton pregnancies and intact membranes who were admitted with a diagnosis of PTL between 23 and 34 weeks' gestation. Pregnancies were eligible for inclusion if amniocentesis was performed at admission and ruled out the occurrence of IAI. Exclusion criteria included latency to delivery shorter than 4 weeks and delivery < 34 weeks. Fetal echocardiography was performed within 72 h after admission and was repeated at follow‐up 4 weeks later. A control group of singleton pregnancies without PTL or preterm prelabor rupture of membranes (PPROM), matched with the study group at a ratio of 1:1 by gestational age at ultrasound, was also evaluated. Fetal cardiac morphology and function were assessed using a standardized echocardiographic protocol, and compared between the two groups. Amniotic fluid troponin I and N‐terminal pro‐brain natriuretic peptide (NT‐proBNP) concentrations were measured in the study group at the time at which amniocentesis was performed, and were compared with 20 amniotic fluid samples from the Clinic‐IDIBAPS Biobank that had been collected for indications other than PTL/PPROM or cardiac pathology.

**Results:**

A total of 32 fetuses exposed to threatened PTL (of which 84.4% were born at term) and 43 controls were compared. At admission, fetuses exposed to threatened PTL already showed signs of cardiac remodeling compared with controls, including a more globular cardiac shape (median global sphericity index, 1.17 *vs* 1.23; *P* = 0.012), an increased right atrial area‐to‐heart area ratio (median, 0.18 *vs* 0.16; *P* = 0.006) and early signs of diastolic dysfunction (median lower tricuspid E/A ratio, 0.65 *vs* 0.72; *P* = 0.027) and increased systolic function (median tricuspid annular plane systolic excursion, 6.5 *vs* 6.0 mm; *P* = 0.036). At admission, the threatened‐PTL group also showed significantly higher amniotic fluid troponin‐I levels (median, 1222.8 pg/mL *vs* 841.3 pg/mL; *P* = 0.026). Amniotic fluid NT‐proBNP was detectable in 25% of these cases, compared with none in the control group. At 4 weeks after the first ultrasound evaluation, in fetuses from mothers with threatened PTL, we observed that systolic function had normalized but cardiac remodeling persisted. These fetuses still exhibited a more globular cardiac shape (median global sphericity index, 1.16 *vs* 1.25; *P* = 0.036), and they now had a larger right ventricle (RV) (median RV area‐to‐heart area ratio, 0.26 *vs* 0.24; *P* = 0.037) without cardiomegaly. Diastolic function remained altered, with prolonged tricuspid inflow duration (median inflow time fraction, 0.39 *vs* 0.35; *P* = 0.001) and longer atrial contraction (median atrial time fraction, 0.23 *vs* 0.20; *P* = 0.039).

**Conclusions:**

Fetuses exposed to threatened PTL without IAI, who were born ≥ 34 weeks' gestation, exhibited signs of cardiac remodeling and subclinical diastolic dysfunction of the RV at admission that persisted for at least 4 weeks. Early identification of threatened PTL may provide opportunities to apply preventive strategies aimed at improving long‐term cardiovascular health. © 2026 The Author(s). *Ultrasound in Obstetrics & Gynecology* published by John Wiley & Sons Ltd on behalf of International Society of Ultrasound in Obstetrics and Gynecology.

## INTRODUCTION

Preterm labor (PTL) is defined as the presence of regular uterine contractions before 37 weeks' gestation with intact membranes associated with progressive cervical shortening and/or cervical dilation. Despite advances in the prediction of preterm delivery^1^, ^2^, around 50% of patients who are admitted with a diagnosis of PTL and intact membranes ultimately deliver near or at term^3^, ^4^, ^5^. Such cases in which the episode of PTL does not progress to preterm delivery are referred to as threatened PTL and account for approximately 1% of term deliveries.

Despite this apparently favorable outcome, children with a history of threatened PTL have an increased risk of low birth weight^6^, ^7^ and adverse neurodevelopmental outcomes, including impairments in motor function, cognition, memory, speed processing, receptive language and affective domains^8^, ^9^. This suggests that the PTL process itself may have direct fetal consequences beyond gestational age at delivery. The cardiovascular profile of these fetuses represents an area of growing interest^10^, yet it has not been characterized previously.

Data regarding fetal cardiovascular programming in PTL are scarce and most of the available data are related to exposure to intra‐amniotic infection or inflammation (IAI) or preterm prelabor rupture of membranes (PPROM)^11^, ^12^, ^13^. Previously, we have reported that fetuses with IAI in a cohort of patients with PTL and/or PPROM presented concentric myocardial hypertrophy and subclinical diastolic dysfunction, as well as increased amniotic fluid (AF) concentrations of N‐terminal pro‐brain natriuretic peptide (NT‐proBNP), as a marker of cardiac overload, and troponin I, as a biomarker of cardiac injury^14^. These changes were in line with postnatal remodeling described in premature infants, who have increased incidence of heart failure^15^, ischemic heart disease^16^ and hypertension^17^. Similarly, albeit more subtly, in the same study we also observed a pattern of cardiac remodeling in fetuses without IAI, which typically have a longer latency to delivery. Notably, that study group included pregnancies with threatened PTL^14^.

To our knowledge, it remains unknown whether these fetuses with threatened PTL present signs of cardiac remodeling and dysfunction. This might be clinically relevant, since these patients are currently managed as low‐risk patients without any fetal or postnatal cardiac evaluation. Therefore, this study aimed to evaluate fetal cardiovascular changes at admission and 4 weeks after the episode in patients with threatened PTL without IAI who delivered ≥ 34 weeks.

## METHODS

This was a prospective cohort study conducted in accordance with the Strengthening the Reporting of Observational Studies in Epidemiology (STROBE) guidelines for study design and reporting^18^. The research ethics committees of the Hospital Clinic and Hospital Sant Joan de Déu reviewed and approved the study (HCB/2018/0567 and PIC 150‐19, respectively) and all participants were informed and provided signed written consent.

### Study population and design

#### Study groups

The study group included consecutive singleton pregnant patients admitted to one of two tertiary referral centers (BCNatal: Hospital Clinic or Hospital Sant Joan de Déu, Barcelona, Spain) between September 2018 and April 2021 with a diagnosis of PTL and intact membranes between 23 + 0 and 34 + 0 weeks' gestation. Patients underwent amniocentesis at admission to rule out the occurrence of IAI.

A group of outpatient singleton pregnant women without PTL or PPROM were also recruited as a control group, matched by gestational age at study ultrasound examination at a ratio of 1:1 with the threatened‐PTL study cases. Amniocentesis was not performed in the control group because there was no clinical indication for it. Instead, in order to compare AF biomarkers of cardiovascular dysfunction, 20 AF samples from the Clinic‐IDIBAPS Biobank that had been collected for indications other than PTL/PPROM or cardiac pathology were selected (Biobank AF samples). Amniocentesis to obtain these samples had been performed in the second or third trimester of pregnancy.

#### Exclusion criteria

Exclusion criteria were delivery within the first 4 weeks after admission, delivery < 34 weeks' gestation, maternal age under 18 years, multiple gestation, clinical chorioamnionitis at admission^19^, and major fetal structural malformation or chromosomal anomaly.

#### Definitions

PTL was defined as regular uterine contractions with a cervical length below the 5^th^ centile^20^ at transvaginal ultrasound examination. We evaluated cervical changes by digital examination only if imminent delivery was suspected. Threatened PTL was defined as an episode of PTL without IAI, with a latency period to delivery of more than 4 weeks and with delivery occurring after 34 weeks.

Intra‐amniotic infection was defined as a positive AF culture for genital mycoplasma (Mycoplasma IST 2 diagnostic kit (bioMérieux, Marcy‐l'Étoile, France) for *Mycoplasma hominis* or *Ureaplasma* spp), or aerobic (cultured on chocolate agar) or anaerobic (cultured on Schaedler agar and in thioglycolate broth) bacteria. In some cases, intra‐amniotic infection was also diagnosed based on specific polymerase chain reaction amplification of the 16S ribosomal RNA gene using the primers: 5′‐AGA GTT TGA TCC TGG CTC AG‐3′ and 5′‐GGA CTA CCA GGG TAT CTA AT‐3′ followed by Sanger sequencing. Sequences were identified using the Blast algorithm in the National Center for Biotechnology Information database^21^, with a minimum of 98% of sequence identity. Intra‐amniotic inflammation was defined by the occurrence of high levels of AF interleukin (IL)‐6, with a cut‐off of 13.4 ng/mL for PTL cases^2^, as reported previously by our group. AF IL‐6 levels were measured using an enzyme‐linked immunosorbent assay (Diasource ImmunoAssays, Louvain‐la‐Neuve, Belgium), with a minimum detection level of 2 pg/mL. The coefficient of variation was 6.23% for a mean concentration of 123.3 pg/mL and 5.18% for a mean concentration of 317.4 pg/mL. Gestational age in weeks was calculated according to first‐trimester crown–rump length^22^.

#### Clinical management of PTL/PPROM

Two intramuscular injections of 12 mg betamethasone given 24 h apart were administered for fetal lung maturation at the time of admission in all cases of threatened PTL. Repeated doses of betamethasone were administered only on suspicion of a high risk of imminent delivery before 34 completed weeks. In the absence of any clinical contraindication, tocolysis with Atosiban or Nifedipine was given during steroid administration. We administered broad‐spectrum antibiotics only to women with suspected intra‐amniotic infection based on the presence of AF glucose concentrations < 5 mg/dL and/or with microorganisms identified by Gram staining and/or positive cultures.

### Fetal echocardiography

Fetal echocardiography was performed in the study group within the first 72 h after admission for the diagnosis of PTL, and was subsequently repeated 4 weeks later to assess the transience or persistence of cardiac changes. The results were compared with those of the control group, which underwent both echocardiographic evaluations at comparable gestational ages.

Echocardiography was performed using Voluson E10 or Voluson S8 ultrasound equipment (GE Healthcare, Zipf, Austria) by an experienced sonographer (C.M. or F.C.). The study protocol included assessment of estimated fetal weight (EFW)^23^, conventional fetoplacental Doppler, and a complete two‐dimensional, M‐mode and Doppler echocardiographic examination to assess cardiac structure, morphometry and function, following a strict, standardized methodology^24^, ^25^, ^26^, ^27^. Fetal cardiac morphometry included the cardiothoracic areas and cardiac sphericity, right and left ventricular areas and sphericities, atrial areas, septal thickness and myocardial thickness of the right and left ventricular free walls. Fetal cardiac systolic function was evaluated using tricuspid annular plane systolic excursion (TAPSE) and mitral annular plane systolic excursion (MAPSE), left ventricular shortening fraction, right ventricular fractional area change, stroke‐volume indices and cardiac outputs and left ejection and isovolumetric contraction time. Fetal cardiac diastolic function was evaluated by atrioventricular peak velocities at early diastole (E) and atrial contraction (A), E/A ratios, tricuspid and mitral inflow time fraction and left isovolumetric relaxation time. The details of each measurement are described in Table S1.

### Cardiac biomarkers in amniotic fluid

A total of 500 μL of AF was collected and frozen at –80°C. All samples were thawed and immediately centrifuged at 16000 × *g* for 4 min prior to use or dilution. Total protein concentrations were evaluated using the Pierce™ BCA Protein Assay Kit (ref: 23225; Thermo Scientific, Waltham, MA, USA) to estimate the appropriate dilution factor for Luminex assays. Samples were analyzed in duplicate and diluted as follows: 1/1 and 1/2 for NT‐proBNP; 1/5 and 1/10 for troponin I. NT‐proBNP was measured using the Human NT Pro‐BNP DuoSet ELISA assay (DY3604‐05; R&D Systems, Minneapolis, MN, USA). Troponin I was measured using a Human Luminex® Discovery Assay (LXSAHM‐04; R&D Systems). A Luminex 100/200™ analyzer (LX100; serial number LX10010187403; Luminex Corp., Austin, TX, USA) and xPONENT® software (Luminex Corp.) were used for 96‐well MagPlex®‐based analyses (MagPlex® microspheres/plates; Luminex Corp.), with a minimum threshold of 50 events. Seven standards with a 1/3 dilution factor were used to perform the calibration curve from a stock solution with a concentration of 12 150 pg/mL for troponin I and 10 000 pg/mL for NT‐proBNP. The lower limit of NT‐proBNP detection was 250 pg/mL. According to the manufacturer's assay specifications, the lower limit of detection for troponin I was 14.9 pg/mL. In our calibration curve, the lowest standard concentration was 16.7 pg/mL. All procedures were performed following the manufacturer's recommendations.

### Statistical analysis

All patient‐identifying information was removed and each participant was assigned a unique study code. The coded clinical and biomarker data were then entered into and managed in a Microsoft Access database (Microsoft Corp., Redmond, WA, USA). Qualitative variables are given as *n* (%) and quantitative variables as median (interquartile range (IQR)). For the baseline, fetal, perinatal and admission characteristics of the study population, univariable analysis was performed using the chi‐square or Fisher's exact test for comparison of qualitative variables. For quantitative variables, Student's *t*‐test was used for independent samples if normal distribution was confirmed by the Shapiro–Wilk test and homoscedasticity by the Levene test. The Wilcoxon‐W test was applied for variables with a non‐normal distribution. For the analysis of echocardiographic and AF biomarker variables, multivariable analysis was performed using multiple linear regression for continuous variables and logistic regression for categorical variables, controlling for potential confounding factors. Echocardiographic variables were adjusted for EFW < 10^th^ centile and AF biomarkers were adjusted for gestational age at amniocentesis. Additionally, an echocardiographic and biomarker subanalysis limited to term deliveries was performed as a sensitivity analysis. Data were analyzed using STATA for MAC version 15.1 (StataCorp LLC, College Station, TX, USA). *P* ≤ 0.05 was considered statistically significant.

## RESULTS

### Study population

**Figure 1:**
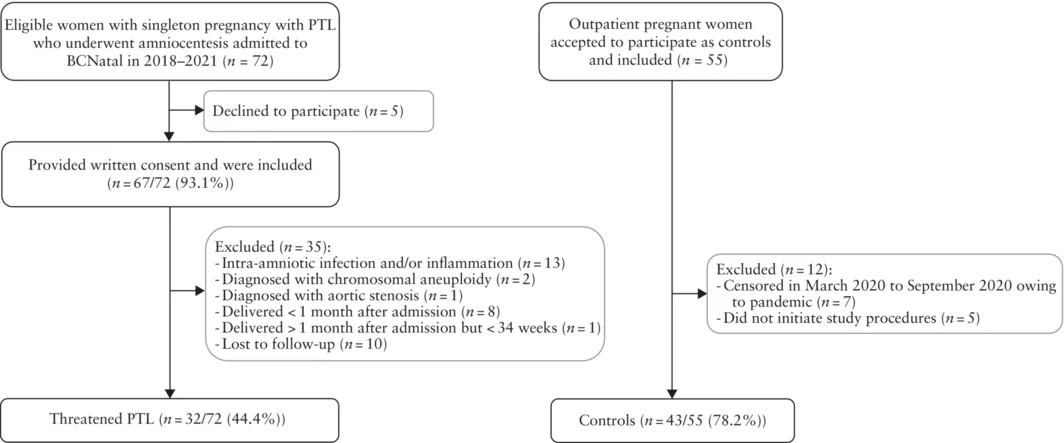
Flowchart summarizing recruitment of study population and controls. BCNatal, Barcelona Center for Maternal‐Fetal and Neonatal Medicine (Hospital Clinic and Hospital Sant Joan de Déu); PTL, preterm labor.

During the study period (2018 to 2021), 83 women with a singleton pregnancy were admitted for PTL between 23 + 0 and 34 + 0 weeks' gestation. Of these, 72 underwent amniocentesis at admission. After exclusions and losses to follow‐up, 32 patients who underwent both echocardiographic examinations (at admission and after 4 weeks) were included in the study group (Figure 1). Approximately 80% of these women admitted for threatened PTL had previously been included in other non‐interventional studies^1^, ^28^. A total of 43 pregnancies were included as the control group.

There were no significant differences in maternal characteristics between the two groups, except for a slightly lower maternal age in the threatened‐PTL group (Table 1). Only one patient with threatened PTL had pregestational hypertension. Two cases of mild pre‐eclampsia were diagnosed in each group and there was one case of gestational diabetes mellitus after inclusion in the control group. No case of pregestational diabetes mellitus or human immunodeficiency virus positivity was reported.

**Table 1:** Baseline, fetal and perinatal characteristics of 32 singleton pregnancies with threatened preterm labor (PTL) and 43 gestational‐ age‐matched controls

Variable	Threatened PTL (*n* = 32)	Controls (*n* = 43)	*P*
Maternal characteristics			
Maternal age (years)	32.5 (27.3–34.8)	33.9 (31.0–37.0)	0.014
Maternal BMI (kg/m^2^)	22.3 (20.3–25.2)	22.1 (21.0–27.7)	0.470
White ethnicity	23 (71.9)	37 (86.0)	0.076
Maternal smoking	4 (12.5)	5 (11.6)	1.000
Nulliparous	23 (71.9)	24 (55.8)	0.105
Assisted reproductive technology	1 (3.1)	5 (11.6)	0.392
Pregnancy and delivery data			
GA at inclusion (weeks)	29.4 (26.4–31.0)	29.9 (26.7–31.4)	0.586
Time from inclusion to delivery (days)	64.0 (51.0–79.0)	71.5 (59.0–85.0)	0.124
Male sex	22 (68.8)	20 (46.5)	0.055
GA at delivery (weeks)	38.6 (37.3–39.7)	39.6 (38.9–40.3)	0.001
Preterm delivery 34 + 0 to 36 + 6 weeks	5 (15.6)	0 (0)	0.012
Birth weight (g)	2980 (2502–3140)	3280 (3000–3510)	0.015
Birth weight < 10^th^ centile	7 (21.9)	6 (14.0)	0.243
Cesarean section	5 (15.6)	8 (18.6)	0.992
5‐min Apgar score < 7	0 (0)	0 (0)	NA
Umbilical artery pH < 7.10	2 (6.3)	4 (9.3)	0.635
Neonatal outcome			
NICU admission	1 (3.1)	0 (0)	0.414
Major neonatal morbidity* or mortality	1 (3.1)	0 (0)	0.414

Data are presented as median (interquartile range) for quantitative variables and *n* (%) for qualitative variables.

* Major neonatal morbidity defined by the presence of bronchopulmonary dysplasia, necrotizing enterocolitis, intraventricular hemorrhage, periventricular leukomalacia, retinopathy or early‐onset sepsis. BMI, body mass index; GA, gestational age; NA, not applicable; NICU, neonatal intensive care unit.

At admission, patients in the threatened‐PTL group had a short cervical length (median, 13 (IQR, 10–19) mm), normal AF glucose concentrations (median, 38 (IQR, 29–47) mg/dL) and low AF IL‐6 concentrations (median, 1060.6 (IQR, 728.5–1698.6) pg/mL). Antenatal corticosteroids were administered for lung maturation in 100% of patients with threatened PTL and in 0% of the control group, an average of two doses per patient being administered.

As expected, controls presented a higher gestational age at delivery and higher birth weight compared with patients with threatened PTL (Table 1). The majority of deliveries in the threatened‐PTL group occurred at term (27/32 (84.4%)). In this group, there was one case with major neonatal morbidity requiring admission to the neonatal intensive care unit, in the context of intrapartum hypoxic–ischemic encephalopathy due to placental abruption. No case of neonatal mortality was reported. There were no other significant differences between the groups. Table S2 shows baseline parameters limited to term deliveries.

### First ultrasound examination (at admission)

The first echocardiographic assessment was performed after administration of at least the first dose of corticosteroids, and in 58% of cases after completion of the full course, always within 48 h of the second dose. There were no differences between the group with threatened PTL and the control group in gestational age, EFW, EFW centile or fetoplacental Doppler findings (Table 2).

**Table 2:** Fetal and placental parameters at admission and at 4 weeks follow‐up in 32 singleton pregnancies with threatened preterm labor (PTL) and 43 gestational‐age‐matched controls

	At admission (1^st^ US)			4 weeks after admission (2^nd^ US)		
Parameter	Threatened PTL	Controls	*P*	Threatened PTL	Controls	*P*
GA at US (weeks)	29.7 (26.7–31.3)	29.9 (26.7–31.4)	0.900	34.1 (32.1–35.8)	34.0 (31.0–36.0)	0.677
EFW at US (g)	1402.5 (1060.0–1766.0)	1378.0 (1079.0–1766.0)	0.988	2247.5 (1750.5–2611.5)	2190.5 (1860.0–2482.0)	0.970
EFW centile	63.5 (37.0–64.0)	52.0 (22.0–69.0)	0.313	52.0 (17.0–77.5)	50.0 (23.0–81.0)	0.458
SGA (EFW < p10)	1 (3.1)	4 (9.3)	0.386	7 (21.9)	4 (9.3)	0.188
UA‐PI	0.97 (0.82–1.11)	1.00 (0.79–1.14)	0.416	0.87 (0.77–1.00)	0.96 (0.84–1.04)	0.173
MCA‐PI	1.78 (1.62–2.10)	2.00 (1.73–2.25)	0.248	1.77 (1.52–2.02)	1.92 (1.67–2.12)	0.352
MCA‐PSV (cm/s)	33.8 (28.4–41.0)	37.9 (33.1–45.0)	0.069	47.6 (42.0–56.2)	45.1 (39.5–58.0)	0.696
CPR	2.07 (1.54–2.40)	1.93 (1.67–2.52)	0.413	2.04 (1.81–2.38)	2.09 (1.64–2.33)	0.567
DV‐PI	0.52 (0.34–0.58)	0.44 (0.35–0.60)	0.870	0.52 (0.26–0.60)	0.51 (0.37–0.61)	0.397
Mean UtA‐PI	0.75 (0.62–0.90)	0.71 (0.64–0.83)	0.495	0.75 (0.62–0.86)	0.72 (0.54–0.84)	0.346

Data are presented as median (interquartile range) for quantitative variables and *n* (%) for qualitative variables. *P* adjusted for estimated fetal weight (EFW) < 10^th^ centile (p10). CPR, cerebroplacental ratio; DV, ductus venosus; GA, gestational age; MCA, middle cerebral artery; PI, pulsatility index; PSV, peak systolic velocity; SGA, small‐for‐gestational age; UA, umbilical artery; US, ultrasound; UtA, uterine artery.

**Figure 2:**
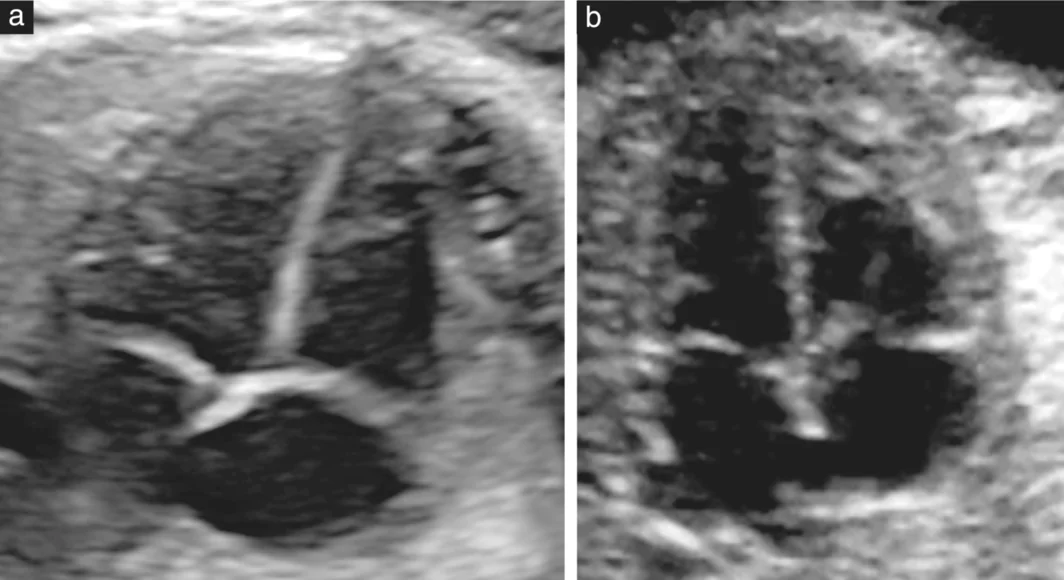
Cardiac morphology in 29 + 6‐week fetus exposed to threatened preterm labor (a) and normal control fetus at 30 + 5 weeks' gestation (b): four‐chamber views obtained by transabdominal two‐dimensional grayscale ultrasound imaging in axial plane, showing more spherical cardiac shape in fetus with threatened preterm labor.

With regard to the fetal echocardiographic findings at admission (Tables 3, 4, 5), fetuses with threatened PTL exhibited signs of cardiac remodeling, with a more globular cardiac morphology, characterized by a reduced global sphericity index, primarily due to remodeling of the right ventricle (RV), without evidence of myocardial hypertrophy or cardiomegaly (Figure 2). The area of the right atrium was also significantly enlarged in these fetuses. With regard to systolic function, fetuses with threatened PTL showed increased TAPSE and an elevated RV stroke‐volume index, despite an absence of significant differences in overall cardiac output or heart rate. Assessment of diastolic function showed a reduced tricuspid E/A ratio of the RV, along with prolonged diastole, mainly due to a lengthened A‐wave. No significant differences were detected in left‐heart parameters. The findings were mostly similar when the analysis was restricted to term deliveries (Tables S3–S6).

**Table 3:** Global parameters: fetal echocardiographic results at admission and at 4 weeks follow‐up in 32 singleton pregnancies with threatened preterm labor (PTL) and 43 gestational‐age‐matched controls

	At admission (1^st^ US)			4 weeks after admission (2^nd^ US)		
Parameter	Threatened PTL	Controls	*P*	Threatened PTL	Controls	*P*
Cardiothoracic ratio (%)*	26.1 (24.1–29.9)	28.6 (26.4–31.5)	0.105	28.4 (26.2–30.5)	28.6 (26.2–31.3)	0.173
Global cardiac sphericity index†	1.17 (1.11–1.22)	1.23 (1.15–1.30)	0.012	1.16 (1.07–1.27)	1.25 (1.14–1.30)	0.036
Septal thickness (mm)	3.8 (2.9–4.2)	3.5 (3.1–3.9)	0.161	4.4 (3.6–4.8)	4.1 (3.5–4.9)	0.463
Heart rate (bpm)	141 (136–152)	145 (139–152)	0.211	141 (135–145)	146 (138–150)	0.060
Cardiac index (mL/min/kg)‡	489.4 (374.3–588.9)	434.7 (383.3–513.7)	0.295	428.8 (338.9–470.3)	434.0 (348.3–496.9)	0.883
RV/LV basal diameter ratio	1.08 (1.01–1.11)	1.06 (0.91–1.16)	0.687	1.07 (0.93–1.20)	1.07 (0.99–1.34)	0.308
Pulmonary artery/aorta diameter ratio	1.22 (1.15–1.47)	1.18 (1.07–1.35)	0.087	1.23 (1.11–1.44)	1.22 (1.10–1.35)	0.033

Data are presented as median (interquartile range). *P* adjusted for estimated fetal weight < 10^th^ centile.

* Cardiothoracic ratio = (cardiac area (cm^2^)/thoracic area (cm^2^)) × 100.

† Cardiac sphericity index = cardiac longitudinal diameter (mm)/transverse diameter (mm).

‡ Cardiac index = (right cardiac output (mL/min) + left cardiac output (mL/min))/estimated fetal weight (kg). LV, left ventricle; RV, right ventricle; US, ultrasound.

**Table 4:** Right heart: fetal echocardiographic results at admission and at 4 weeks follow‐up in 32 singleton pregnancies with threatened preterm labor (PTL) and 43 gestational‐age‐matched controls

	At admission (1^st^ US)			4 weeks after admission (2^nd^ US)		
Parameter	Threatened PTL	Controls	*P*	Threatened PTL	Controls	*P*
Right cardiac morphometry						
RA area‐to‐heart area ratio*	0.18 (0.16–0.21)	0.16 (0.13–0.18)	0.006	0.17 (0.14–0.20)	0.16 (0.14–0.18)	0.087
RV area‐to‐heart area ratio*	0.23 (0.20–0.28)	0.25 (0.20–0.29)	0.583	0.26 (0.21–0.28)	0.24 (0.20–0.25)	0.037
RV sphericity index†	1.63 (1.47–1.81)	1.73 (1.56–1.93)	0.085	1.61 (1.39–1.83)	1.76 (1.60–1.93)	0.036
RV myocardial free‐wall thickness (mm)	2.8 (2.4–3.7)	3.1 (2.6–3.3)	0.558	3.5 (3.0–3.9)	3.5 (3.0–3.8)	0.387
Right systolic function						
TAPSE (mm)	6.5 (5.8–7.6)	6.0 (5.4–6.7)	0.036	7.0 (6.4–7.9)	7.1 (6.4–7.6)	0.632
Right FAC (%)‡	33.8 (30.2–41.7)	36.9 (28.1–48.1)	0.564	32.4 (26.9–44.9)	35.4 (29.8–43.3)	0.400
Pulmonary artery diameter (mm)	6.1 (5.4–6.9)	5.6 (5.1–6.7)	0.153	7.7 (7.0–8.2)	7.0 (6.5–8.0)	0.049
Pulmonary artery PSV (cm/s)	68.0 (62.2–80.6)	66.6 (59.6–74.8)	0.344	69.3 (58.7–78.2)	74.2 (61.1–86.0)	0.107
Right SVI (mL/kg)§	1.93 (1.15–2.57)	1.78 (1.47–2.00)	0.045	1.81 (1.41–2.32)	1.87 (1.45–2.09)	0.696
Right cardiac output index (mL/min/kg)¶	286 (216–376)	254 (217–296)	0.072	260 (192–316)	270 (201–295)	0.891
Right diastolic function						
Tricuspid E/A ratio	0.65 (0.63–0.73)	0.72 (0.62–0.73)	0.027	0.74 (0.68–0.83)	0.75 (0.70–0.80)	0.483
Tricuspid inflow time fraction**	0.39 (0.37–0.43)	0.38 (0.34–0.41)	0.103	0.39 (0.36–0.43)	0.35 (0.32–0.38)	0.001
Tricuspid A (atrial) time fraction**	0.23 (0.21–0.25)	0.21 (0.20–0.23)	0.017	0.23 (0.20–0.25)	0.20 (0.18–0.22)	0.039

Data are presented as median (interquartile range). *P* adjusted for estimated fetal weight < 10^th^ centile.

* Right atrial (RA) or right ventri cular (RV) area‐to‐heart area ratio = RA or RV area (cm^2^)/cardiac area (cm^2^).

† Ventricular sphericity index = ventricular longitudinal diameter (mm)/ transverse diameter (mm).

‡ Fractional area change (FAC) = ((end‐diastolic RV area (cm^2^) − end‐systolic RV area (cm^2^)) /end‐diastolic RV area (cm^2^)) × 100.

§ Stroke volume index (SVI) = (pulmonary artery area (cm^2^) × pulmonary artery velocity time integral (cm/contraction))/estimated fetal weight (kg).

¶ Right cardiac output index = (right (pulmonary) stroke volume (mL/contraction) × heart rate (bpm))/estimated fetal weight (kg).

** Diastolic times fraction = tricuspid inflow or A‐wave duration (ms)/total cycle duration (ms). A, atrio ventricular peak velocity at atrial contraction; E, atrioventricular peak velocity at early diastole; PSV, peak systolic velocity; TAPSE, tricuspid annular plane systolic excursion; US, ultrasound.

**Table 5:** Left heart: fetal echocardiographic results at admission and at 4 weeks follow‐up in 32 singleton pregnancies with threatened preterm labor (PTL) and 43 gestational‐age‐matched controls

	At admission (1^st^ US)			4 weeks after admission (2^nd^ US)		
Parameter	Threatened PTL	Controls	*P*	Threatened PTL	Controls	*P*
Left cardiac morphometry						
LA area‐to‐heart area ratio*	0.17 (0.15–0.19)	0.14 (0.13–0.18)	0.067	0.15 (0.13–0.17)	0.15 (0.13–0.17)	0.943
LV area‐to‐heart area ratio*	0.26 (0.24–0.31)	0.26 (0.22–0.30)	0.328	0.24 (0.22–0.27)	0.24 (0.20–0.26)	0.189
LV sphericity index†	1.70 (1.53–1.89)	1.79 (1.60–1.92)	0.250	1.69 (1.59–1.81)	1.86 (1.63–1.99)	0.056
LV myocardial free‐wall thickness (mm)	3.3 (2.4–3.9)	2.8 (2.4–3.3)	0.113	3.6 (3.0–3.9)	3.6 (2.9–4.1)	0.804
Left systolic function						
MAPSE (mm)	4.2 (3.9–4.7)	4.2 (3.7–5.0)	0.817	5.4 (4.1–6.1)	4.9 (4.0–5.8)	0.315
LV shortening fraction (%) ‡	0.42 (0.35–0.51)	0.40 (0.34–0.48)	0.735	0.44 (0.33–0.49)	0.39 (0.31–0.45)	0.118
Left ICT index (%)§	8.7 (7.8–9.3)	8.1 (6.8–9.4)	0.113	8.5 (7.3–9.0)	7.6 (6.7–9.5)	0.345
Left ET index (%)§	40.1 (38.7–42.7)	39.9 (38.5–42.0)	0.986	37.9 (36.8–39.6)	39.0 (37.3–40.9)	0.174
Aortic diameter (mm)	4.6 (4.3–5.2)	4.9 (4.4–5.4)	0.557	5.7 (5.3–6.8)	6.0 (4.9–6.8)	0.962
Aortic PSV (cm/s)	81.4 (77.2–90.8)	78.7 (70.8–93.6)	0.978	84.0 (73.3–94.0)	84.9 (74.9–97.8)	0.298
Left SVI (mL/kg)¶	1.31 (0.93–1.68)	1.30 (1.06–1.60)	0.936	1.09 (0.88–1.40)	1.22 (0.95–1.68)	0.741
Left cardiac output index (mL/min/kg)**	184 (148–220)	187 (159–244)	0.680	145 (126–204)	169 (133–232)	0.527
Left diastolic function						
Mitral E/A ratio	0.71 (0.67–0.75)	0.73 (0.68–0.79)	0.202	0.75 (0.69–0.84)	0.72 (0.68–0.82)	0.051
Mitral inflow time fraction††	0.42 (0.39–0.45)	0.43 (0.40–0.45)	0.899	0.44 (0.40–0.46)	0.40 (0.36–0.45)	0.059
Mitral A (atrial) time fraction††	0.22 (0.21–0.24)	0.22 (0.20–0.24)	0.592	0.23 (0.20–0.25)	0.22 (0.20–0.24)	0.135
Left IRT index (%)§	10.9 (10.1–11.8)	11.9 (10.4–13.0)	0.173	11.4 (10.9–14.0)	12.0 (11.0–14.0)	0.681
Left combined systolic and diastolic function						
MPI‡‡	0.50 (0.45–0.53)	0.50 (0.44–0.56)	0.867	0.56 (0.47–0.61)	0.52 (0.48–0.58)	0.288

Data are presented as median (interquartile range). *P* adjusted for estimated fetal weight < 10^th^ centile.

* Left atrial (LA) or left ventricular (LV) area‐to‐heart area ratio = LA or LV area (cm^2^)/cardiac area (cm^2^).

† Ventricular sphericity index = ventricular longitudinal diameter (mm)/ transverse diameter (mm).

‡ LV shortening fraction (M‐mode) = ((end‐diastolic LV diameter (mm) − end‐systolic LV diameter (mm))/end‐diastolic LV diameter (mm)) × 100.

§ Isovolumetric contraction time (ICT) index, ejection time (ET) index or isovolumetric relaxation time (IRT) index = (ICT, ET or IRT (ms)/total cycle duration (ms)) × 100.

¶ Stroke volume index (SVI) = (aortic area (cm^2^) × aortic velocity time integral (cm/contraction))/estimated fetal weight (kg).

** Left cardiac output index = (left (aortic) stroke volume (mL/contraction) × heart rate (bpm))/estimated fetal weight (kg).

†† Diastolic times fraction = mitral inflow or A‐wave duration (ms)/total cycle duration (ms).

‡‡ Myocardial performance index (MPI) = (ICT (ms) + IRT (ms))/ET (ms). A, atrioventricular peak velocity at atrial contraction; E, atrioventricular peak velocity at early diastole; MAPSE, mitral annular plane systolic excursion; PSV, peak systolic velocity; PTL, preterm labor; US, ultrasound.

### Second ultrasound examination (4 weeks after admission)

On ultrasound examination 4 weeks after admission, there were no differences between the group with threatened PTL and the control group in gestational age, EFW, EFW centile or fetoplacental Doppler findings (Table 2). However, it was of note that in the control group, the percentage of fetuses with EFW < 10^th^ centile (small‐for‐gestational‐age (SGA) fetuses) remained stable 4 weeks after the first ultrasound examination, at around 9%, whereas it increased from 3% to 22% in the threatened‐PTL group.

Regarding fetal echocardiographic evaluation 4 weeks after admission (Tables 3, 4, 5), fetuses with threatened PTL presented persistence of more globular cardiac morphology at the expense of the RV, without myocardial hypertrophy or cardiomegaly. By this time, the size of the right atrium was normal, although the RV area and the pulmonary artery diameter were slightly increased. With regard to systolic function 4 weeks after admission, all the parameters were normal with no differences between the groups. With respect to diastolic function, signs of impaired RV relaxation persisted, with prolonged tricuspid inflow times and longer atrial contraction. Similar results were obtained when the analysis was restricted to term deliveries (Tables S3–S6).

### Cardiac biomarkers in amniotic fluid

**Figure 3:**
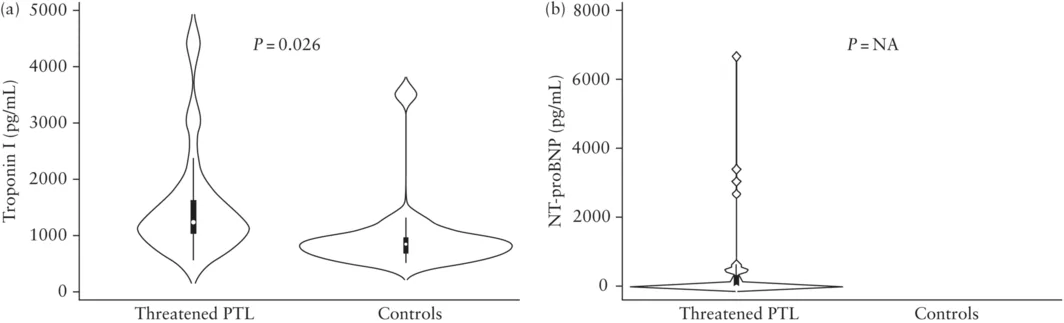
Violin plots showing distribution of cardiac biomarker concentrations in amniotic fluid at admission in 32 singleton pregnancies with threatened preterm labor (PTL) compared with 20 control samples from the Clinic‐IDIBAPS biobank: (a) troponin I, a marker of cardiac injury; (b) N‐terminal pro‐brain natriuretic peptide (NT‐proBNP), a marker of cardiac overload. Embedded boxplots indicate median and interquartile range, and individual points represent single observations. *P* adjusted for gestational age at amniocentesis. NA, not applicable.

Levels of cardiac biomarkers from AF samples taken at admission in fetuses with threatened PTL were compared with those in biobank AF samples. Tables S7 and S8 detail the indications for amniocentesis and the baseline characteristics of this group of biobank samples. Results were therefore adjusted for gestational age at amniocentesis and for EFW < 10^th^ centile. Although NT‐proBNP was detectable in 25% of women with threatened PTL (8/32) and undetectable in all control samples, the difference did not reach statistical significance. The distribution of NT‐proBNP concentrations is shown in Figure 3. AF troponin‐I concentrations were significantly higher in patients with threatened PTL (median, 1222.8 pg/mL *vs* 841.3 pg/mL; *P* = 0.026) compared with those in the control group (Figure 3). Similar findings were observed when the analysis was limited to term deliveries (Table S9).

## DISCUSSION

Our findings indicate that fetuses with threatened PTL show evidence of cardiac remodeling and subclinical dysfunction confined to the right heart, with diastolic and morphological changes persisting at 4‐week follow‐up.

From a pathophysiological perspective, it is noteworthy that fetal cardiac response appears to follow two distinct phases, with exclusive involvement of the RV. At the time of admission, the fetus is likely undergoing an acute adaptive response. The RV shows increased contractility and blood‐volume management (elevated TAPSE and increased RV stroke‐volume index) while adopting a more energy‐efficient, rounded geometry (without cardiomegaly or myocardial hypertrophy) and exhibiting impaired RV diastolic function (reduced E/A ratio and prolonged relaxation times). This pattern is consistent with a hyperdynamic heart under increased pressure load, probably driven by relative hypervolemia, as suggested by the higher stroke‐volume index despite a similar fetal heart rate. As the stimulus is probably acute, the right atrium dilates to accommodate the rise in filling pressure. At this time, higher concentrations of AF troponin I (a marker of cardiac injury) were found, reinforcing the echocardiographic findings.

Four weeks after the episode of PTL, and following clinical stabilization, the acute stimulus had resolved. However, the heart continued to show signs of adaptation, suggesting that another persistent stimulus may have still been contributing to this adaptive response. The better‐adapted RV maintained its more globular configuration, which enhances the efficiency of handling the ongoing pressure overload, allowing normalization of right atrial size and systolic parameters without the need for hypertrophy or cardiomegaly. Both the RV and the pulmonary artery diameter were enlarged, likely reflecting a persistent underlying insult. Subclinical diastolic dysfunction persisted in parallel with this remodeling.

Prenatal data on fetal cardiac assessment in PTL are limited. Aye *et al*.^29^ found no significant cardiac differences between fetuses born preterm and those born at term. However, their study included only preterm deliveries (< 34 weeks) and mixed spontaneous (including IAI) and iatrogenic cases, while our cohort included mostly term deliveries (84%) and were limited to spontaneous PTL (excluding IAI).

Postnatally, preterm infants have been reported to exhibit cardiac remodeling characterized by concentric myocardial hypertrophy and both systolic and diastolic dysfunction^30^, ^31^, ^32^, ^33^. These findings are consistent with previous observations by our group in fetuses with IAI^14^, in which signs of myocardial hypertrophy at admission were observed, and were more pronounced than signs in fetuses without IAI, suggesting that intra‐amniotic inflammation may have been the underlying cause of the cardiac hypertrophy. However, in our current cohort with threatened PTL, the cardiovascular remodeling differed from that observed in our previous study, when the infectious/inflammatory origin was present. These fetuses with threatened PTL, which eventually delivered at or near term, presented a globular heart shape without hypertrophy, unlike the concentric hypertrophy described in the previous IAI group^14^, suggesting the existence of an alternative pathogenic pathway contributing to this remodeling, different from the inflammatory‐related mechanism reported previously by our group.

The type of remodeling observed in our population resembles that described in cases of mild and late‐onset fetal growth restriction (FGR)^34^, ^35^, ^36^. However, growth‐restricted fetuses typically show biventricular changes and a tendency towards reduced systolic performance, which differs from our findings. Furthermore, our results were adjusted for the presence of fetuses with EFW < 10^th^ centile, suggesting that the cardiac remodeling observed was independent of the prevalence of FGR. Nevertheless, it is well documented that approximately 30% of PTL cases exhibit placental dysfunction similar to that seen in FGR^37^, ^38^, ^39^. It is therefore likely that our cohort included such cases, which may explain why some remodeling features resembled FGR patterns rather than those typically reported with IAI. Conversely, the acute contraction‐related stimulus during PTL could transiently augment systolic function, unlike in fetuses with FGR. The fact that changes were limited to the RV in our study are also plausible, given that placental disease in PTL is generally less severe than it is in FGR^40^; thus, the dominant RV may be affected preferentially, being coupled more directly to the placenta.

The increase in the number of SGA fetuses in the threatened‐PTL group at the second ultrasound examination compared with the first ultrasound examination is notable. Further studies are needed to determine whether cardiac remodeling is more pronounced in fetuses that subsequently become SGA compared with those that maintain normal growth until delivery.

As the study excluded fetuses that delivered within 4 weeks of admission, whether earlier delivery is associated with more pronounced cardiac changes at initial assessment remains unknown and warrants further investigation.

The short‐term effects of corticosteroids have been associated with a transient reduction in fetal heart rate, as well as improvements in Doppler parameters and myocardial performance index, without evidence of morphological changes^41^, ^42^. As the main findings in our cohort relate to cardiac morphology, and no differences were observed in these functional parameters, it is unlikely that the observed changes could be attributable to corticosteroid exposure. Furthermore, given the transient nature of these effects and the absence of repeated corticosteroid administration, an influence on the second echocardiographic assessment is also unlikely. Additionally, the evidence available suggests that postnatal cardiac remodeling is independent of corticosteroid exposure^43^, ^44^.

Moderate maternal stress, as may occur during hospitalization for PTL, has been associated with fetal Doppler changes and SGA^45^. Postnatally, maternal perinatal stress has also been linked to septal hypertrophy and systolic and diastolic dysfunction in the neonates of women experiencing such stress^46^. Although Doppler findings and remodeling patterns reported in those studies are not consistent with our current observations, the potential impact of maternal perinatal stress on fetal growth and cardiac function cannot be excluded.

### Strengths and limitations

The main strength of this study lies in its prospective design and the recruitment of a well characterized cohort, making it the first study to provide comprehensive fetal cardiovascular assessment 4 weeks after an episode of threatened PTL.

However, certain limitations should be acknowledged. The sample size was relatively small, which may have led to a lack of statistical significance in some of the outcomes analyzed. Furthermore, the biomarker control group was derived retrospectively from a different population than the prospectively recruited echocardiographic cohorts, which may limit the comparability and interpretation of these results. Echocardiographic measurements were not blinded, which may have introduced observer bias; however, prior evidence supports their reproducibility^27^, ^47^, ^48^, and consistency with blinded biomarker data strengthens the findings. Additionally, it is uncertain whether corticosteroid administration could have influenced our results. Our pathophysiological hypothesis is based on observed data and biological plausibility, and is consistent with the data of previous studies. However, we did not perform placental analysis, and further research is needed to confirm this hypothesis. Our findings are likely applicable to pregnant women with threatened PTL delivering at or beyond 34 weeks' gestation in comparable healthcare settings; extrapolation to earlier gestational ages or different populations should be done with caution. Finally, a key limitation of this study is the lack of postnatal follow‐up. Further research is warranted to evaluate prospectively cardiovascular changes postnatally.

### Conclusions

This study suggests that fetuses exposed to threatened PTL without IAI, delivering at or near term, exhibit signs of persistent fetal cardiovascular remodeling and subclinical diastolic dysfunction of the RV. A transient right systolic adaptation was observed only at admission. The observation of an elevation in the cardiac injury marker AF troponin I reinforces these echocardiographic findings. Further postnatal studies are warranted to determine the persistence of these changes, which may provide a window of opportunity for the early identification of individuals at increased cardiovascular risk.

## Supporting information


**Table S1** Details of fetal echocardiographic measurements.
**Table S2** Baseline, fetal and perinatal characteristics including only deliveries at term (> 37 weeks).
**Table S3** Fetoplacental parameters at admission and at 4‐week follow‐up, including only deliveries at term (> 37 weeks).
**Table S4** Global fetal echocardiographic results at admission and at 4‐week follow‐up including only deliveries at term (> 37 weeks).
**Table S5** Right heart: fetal echocardiographic results at admission and at 4‐week follow‐up including only deliveries at term (> 37 weeks).
**Table S6** Left heart: fetal echocardiographic results at admission and at 4‐week follow‐up including only deliveries at term (> 37 weeks).
**Table S7** Indications for amniocentesis in the Biobank samples.
**Table S8** Baseline characteristics of threatened preterm labor (PTL) *vs* Biobank amniotic fluid sample groups.
**Table S9** Cardiac biomarkers in amniotic fluid at admission including only deliveries at term (> 37 weeks).

## Data Availability

The data that support the findings of this study are available from the corresponding author upon reasonable request.
