# Supplementary material for: Fetuses exposed to an episode of threatened preterm labor delivered at or near term show signs of cardiac remodeling
Source: Ultrasound Obstet Gynecol. 2026 Aug 16;68(3):395–405. doi: 10.1002/uog.70304 (PMC13532152; doi:10.1002/uog.70304)
Supplement: Supplementary file 1 [file UOG-68-395-s001.docx]

| **TABLE S1: Details of fetal echocardiographic measurements** | | |
| --- | --- | --- |
| Variables | Methodology | Example |
| Cardiac morphometry | | |
| Cardiac and thoracic areas  Global cardiac sphericity | Cardiac area was measured in 2D images at maximal distension from an apical or basal 4-chamber view at end-diastole. End-diastole was defined as the frame at which the atrioventricular valves closed and, thus, when the ventricles reached their largest size.  Using the same cardiac area tracing, the longitudinal and transverse cardiac diameters were measured, and cardiac sphericity was calculated as the ratio between the transverse and longitudinal diameters.  Thoracic area was measured in the same image as the cardiac area. |  |
| Atrial areas | Atrial areas were measured in 2D images at maximum atrial distension from a 4-chamber view at end-systole (defined by the frame preceding the atrioventricular valves opening) and, thus, when the atria reached their largest size. The atrial measurements did not include the pulmonary veins/arteries or the atrioventricular valve annulus. |  |
| Ventricular areas and sphericities | Ventricular dimensions and areas were measured in 2D images from an apical or basal 4-chamber view at end-diastole. The ventricular basal and longitudinal dimensions were measured at the level of the atrioventricular valves and from the  atrioventricular valves (including the atrioventricular valves annulus) to the inner myocardium apex, respectively. Both ventricular areas were measured by manual tracing along the true border of the inner myocardium, including the endocardium, the  muscular trabeculations, and the moderator band. |  |
| Myocardial free-wall and septal thicknesses (mm) | Myocardial wall thicknesses were measured in 2D images from a transverse 4-chamber view at end-diastole below the atrioventricular valve leaflets. |  |
| Systolic function | | |
| TAPSE, MAPSE | An apical or basal four-chamber view was used, applying M-mode at the tricuspid (TAPSE) or mitral (MAPSE) valve annu­lus to measure the maximum excursion from end-diastole to end-systole. |  |
| End-systolic septal thickness (used for calculating septal thickening) | Septal thickness was measured in 2D images from a transverse 4-chamber view at end-systole. |  |
| End-systolic right ventricular area (used for calculating the right fractional change area (FAC)) | End systolic right ventricular area was measured in 2D images from an apical or basal 4-chamber view at end-systole by manual tracing along the true border of the inner myocardium, including the endocardium, the muscular trabeculations, and the moderator band. |  |
| Mid diameter of ventricular cavity (used for calculating left ventricular shortening fraction) | Left mid-ventricular cavity was measured at end-systole and end-diastole using M mode images from a transverse 4-chamber view. |  |
| Left isovolumetric contraction time (ICT) | Placing the Doppler sample volume in a four-cham­ber view on the medial wall of the ascending aorta and displaying the mitral biphasic inflow and the aortic out­flow in the same spectral image. ICT includes the time interval from the closure of the mitral valve to the opening of the aortic valve. |  |
| Left ejection time (ET) | Placing the Doppler sample volume in a four-cham­ber view on the medial wall of the ascending aorta and displaying in the same spectral image the mitral biphasic inflow and the aortic out­flow. Ejection time includes the time interval from the opening to closure of the aortic valve. |  |
| Aortic and pulmonary diameters (mm) | Aortic and pulmonary diameters were obtained in frozen real-time images during early to mid-systole by the leading-edge-to-edge method. |  |
| Aortic and pulmonary velocity-time integrals (used for calculating left and right stroke volume, respectively) | Velocity-time integrals were calculated by manual trace of the spectral Doppler area and can be multiplied by the area of the outflow wave. |  |
|  |  |   |
| Diastolic function | | |
| Tricuspid and mitral E/A ratios | Evaluated by the spectral Doppler sample volume below the atrioventricular valves, displaying a biphasic wave. The ratio is obtained by the division of the peak velocities of the E over the A waveform. |  |
| Tricuspid and mitral inflow time. | Inflow times were evaluated by the spectral Doppler sample volume below the atrioventricular valves, displaying a biphasic wave. Inflow time includes the time interval from the opening to closure of the atrioventricular valves. |  |
| Tricuspid and mitral A waves duration | A wave duration was evaluated by the spectral Doppler sample volume below the atrioventricular valves, displaying a biphasic wave. A duration includes the time interval of the A wave (second wave). |  |
| Left isovolumetric relaxation time (IRT) | Placing the Doppler sample volume in a four-cham­ber view on the medial wall of the ascending aorta and displaying the mitral biphasic inflow and the aortic out­flow in the same spectral image. IRT includes the time interval from the closure of the aortic valve to the opening of the mitral valve. |  |

| **TABLE S2: Baseline, fetal and perinatal characteristics including only deliveries at term (>37 weeks)** | | | |
| --- | --- | --- | --- |
| Variables | Threatened PTL (n= 27) | Control (n=43) | p |
| *Maternal characteristics* | | | |
| Maternal age (years) | 32.3 (26.7-34.8) | 33.9 (31.0- 37.0) | **0.015** |
| BMI (Kg/m^2^) | 22.2 (20.3- 24.9) | 22.1 (21.0- 27.7) | 0.419 |
| Caucasian ethnicity, n (%) | 18 (66.7) | 37 (86.1) | 0.080 |
| Maternal smoking, n(%) | 3 (11.1) | 5 (11.6) | 0.920 |
| Nulliparity, n(%) | 20 (74.1) | 24 (55.8) | 0.077 |
| Assisted Reproductive Technology, n(%) | 1 (3.7) | 5 (11.6) | 0.089 |
| *Pregnancy and delivery data* | | | |
| GA at inclusion (weeks) | 29.4 (26.3- 31.4) | 29.9 (26.7- 31.4) | 0.522 |
| Time from inclusion to delivery (days) | 67.5 (56.0- 81.0) | 71.5 (59.0- 85.0) | 0.595 |
| Neonatal male sex, n(%) | 18 (66.7) | 20 (46.5) | 0.099 |
| GA at delivery (weeks) | 39.0 (37.4- 40.0) | 39.6 (38.9- 40.3) | 0.063 |
| Birthweight (g) | 3065 (2890-3260) | 3280 (3000-3510) | 0.097 |
| Birthweight below 10^th^ percentile, n(%) | 5 (18.5) | 6 (14.0) | 0.612 |
| Cesarean section, n(%) | 3 (11.1) | 8 (18.6) | 0.745 |
| APGAR <7 at 5 min, n(%) | 0 | 0 | NA |
| pH umbilical artery <7.10, n (%) | 1 (3.7) | 4 (9.3) | 0.355 |
| *Neonatal outcome* | | | |
| NICU admission n(%) | 0 | 0 | NA |
| Major neonatal morbidity* or mortality, n(%) | 0 | 0 | NA |
| *Data are presented as number (percentage) for qualitative variables or median (25th centile-75th centile) for quantitative variables.*  *ART, artificial reproduction technique; BMI, body mass index; GA, gestational age; IAI, intra-amniotic infection or inflammation; NICU, neonatal intensive care unit; NA, not applicable; PTL, preterm labor.*  *Major complications defined by the presence of bronchopulmonary dysplasia, necrotizing enterocolitis, intraventricular hemorrhage, periventricular leukomalacia, retinopathy or early onset sepsis. | | | |

| **TABLE S3: Fetoplacental parameters at admission and at 4 weeks follow-up including only deliveries at term (> 37 weeks)** | | | | | | |
| --- | --- | --- | --- | --- | --- | --- |
|  | **First US (at admission)** | | | **Second US (1-month after admission)** | | |
| **Variables** | **Threatened** **PTL (n= 27)** | **Control (n=43)** | **p** | **Threatened** **PTL (n= 27)** | **Control (n=43)** | **p** |
| GA at ultrasound | 2967 (26.7-31.4) | 29.9 (26.7-31.4) | 0.808 | 34.1 (31.5-36.1) | 34.0 (31.0-36.0) | 0.760 |
| EFW at ultrasound (gr) | 1379.0(1060.0-1766.0) | 1378.0(1079.0-1766.0) | 0.999 | 2172.0(1618.0-2660.0) | 2190.5(1860.0-2482.0) | 0.922 |
| EFW centile | 65.0 (41.0-75.0) | 52.0 (22.0-69.0) | 0.228 | 53.0 (9.0-81.0) | 50.0 (23.0-81.0) | 0.475 |
| Small for gestational age (EFW < 10th centile) | 1 (3.7) | 4 (9.3) | 0.355 | 7 (25.9) | 4 (9.3) | 0.066 |
| Umbilical artery PI | 1.01 (0.90-1.12) | 1.00 (0.79-1.14) | 0.965 | 0.88 (0.77-1.04) | 0.96 (0.84-1.04) | 0.269 |
| Middle cerebral artery PI | 1.79 (1.56-2.21) | 2.00 (1.73-2.25) | 0.399 | 1.84 (1.52-2.07) | 1.92 (1.67-2.12) | 0.542 |
| Middle cerebral artery Peak systolic velocity (cm/s) | 34.2 (30.1-41.2) | 37.9 (33.1-45.0) | 0.186 | 50.1 (44.4-56.2) | 45.1 (39.5–58.0) | 0.514 |
| Cerebroplacental ratio | 1.93 (1.67-2.52) | 1.93 (1.67-2.52) | 0.218 | 2.10 (1.64-2.39) | 2.09 (1.64–2.33) | 0.570 |
| Ductus venosus PI | 0.50 (0.33-0.57) | 0.44 (0.35-0.60) | 0.998 | 0.51 (0.37-0.61) | 0.51 (0.37-0.61) | 0.848 |
| Uterine arteries mean PI | 0.75 (0.63-0.90) | 0.71 (0.64-0.83) | 0.319 | 0.75 (0.59-0.85) | 0.72 (0.54-0.84) | 0.409 |
| Data are presented as number (percentage) for qualitative variables or median (25^th^ centile-75^th^ centile) for quantitative variables. **p** was adjusted for estimated fetal weight <10^th^ centile.  *EFW, estimated fetal weight; GA, gestational age; PI, pulsatility index; PTL, preterm labor; US: Ultrasound* | | | | | | |

| **TABLE S4: Global fetal echocardiographic results at admission and at 4 weeks follow-up including only deliveries at term (> 37 weeks)** | | | | | | |
| --- | --- | --- | --- | --- | --- | --- |
| **Variables** | **First US (at admission)** | | | **Second US (1-month after admission)** | | |
|  | **Threatened** **PTL (n= 27)** | **Control (n=43)** | **p** | **Threatened** **PTL (n= 27)** | **Control (n=43)** | **p** |
|  | **Global parameters** | | | **Global parameters** | | |
| Cardiothoracic ratio* (%) | 26.30 (24.05-28.66) | 28.6 (26.4-31.5) | 0.100 | 28.7 (24.5-30.8) | 28.6 (26.2-31.3) | 0.070 |
| Global cardiac sphericity index^†^ | 1.16 (1.08-1.22) | 1.23 (1.15-1.30) | **0.011** | 1.17 (1.07-1.28) | 1.25(1.14-1.30) | **0.048** |
| Septal thickness (mm) | 3.8 (2.9-4.4) | 3.5 (3.1-3.9) | 0.161 | 4.15 (3.4-4.7) | 4.1 (3.5-4.9) | 0.887 |
| Heart rate (bpm) | 139 (136-149) | 145 (139-152) | 0.088 | 140 (134-144) | 146 (138-150) | **0.021** |
| Cardiac index (ml/min/kg)^‡^ | 491.1 (374.3-588.9) | 434.7 (383.3-513.7) | 0.238 | 433.6 (376.9-541.6) | 434.0 (348.3-496.9) | 0.774 |
| Data are presented as number (percentage) for qualitative variables or median (25th centile-75th centile) for quantitative variables. p was adjusted for estimated fetal weight <10th centile.  *PTL: Preterm labor; US: Ultrasound*  * Cardio-thoracic ratio: (cardiac area(cm^2^)/thoracic area (cm2))*100  ^†^ Cardiac Sphericity: cardiac longitudinal diameter (mm)/ transverse diameter (mm)  ^‡^ Cardiac index: (Right cardiac output (ml/min)+ left cardiac output (ml/min)) / EFW (Kg). | | | | | | |

| **TABLE S5: Right heart: fetal echocardiographic results at admission and at 4 weeks follow-up including only deliveries at term (> 37 weeks)** | | | | | | |
| --- | --- | --- | --- | --- | --- | --- |
| **Variables** | **First US (at admission)** | | | **Second US (1-month after admission)** | | |
|  | **Threatened** **PTL (n= 27)** | **Control (n=43)** | **p** | **Threatened** **PTL (n= 27)** | **Control (n=43)** | **p** |
|  | *Right cardiac morphometry* | | | *Right cardiac morphometry* | | |
| Right atrial area to heart ratio* | 0.19 (0.16-0.21) | 0.16 (0.13-0.18) | **0.003** | 0.16 (0.14-0.18) | 0.16 (0.14-0.18) | 0.192 |
| Right ventricular area to heart ratio* | 0.24 (0.22-0.28) | 0.25 (0.20-0.29) | 0.798 | 0.25 (0.21-0.28) | 0.24 (0.20-0.25) | **0.042** |
| Right ventricular sphericity index^†^ | 1.60 (1.45-1.81) | 1.73 (1.56-1.93) | **0.041** | 1.64 (1.39-1.83) | 1.76 (1.60-1.93) | **0.081** |
| Right ventricle wall thickness (mm) | 3.1 (2.5-3.7) | 3.1 (2.6-3.3) | 0.272 | 3.6 (3.1-3.9) | 3.5 (3.0-3.8) | 0.298 |
|  | *Right systolic function* | | | *Right systolic function* | | |
| TAPSE | 6.5 (6.1-7.7) | 6.0 (5.4-6.7) | **0.025** | 7.0 (6.4-7.9) | 7.1 (6.4-7.9) | 0.698 |
| Right fractional area change area (FAC) (%)^‡^ | 33.5 (25.8-36.0) | 36.9 (28.1-48.1) | 0.283 | 33.7 (26.9- 42.8) | 35.4 (29.8-43.3) | 0.321 |
| Pulmonary artery diameter (mm) | 6.2 (5.4-6.9) | 5.6 (5.1-6.7) | 0.200 | 7.8 (7.0-8.2) | 7.0 (6.5-8.0) | **0.014** |
| Peak systolic pulmonary artery velocity (cm/s) | 66.1 (62.2-81.4) | 66.6 (59.6-74.8) | 0.369 | 70.0 (64.6-78.2) | 74.2 (61.1-86.0) | 0.158 |
| Right stroke volume index (ml/kg)^§^ | 1.96 (1.15-2.57) | 1.78 (1.47-2.00) | **0.035** | 1.98 (1.69-2.33) | 1.87 (1.45-2.09) | 0.307 |
| Right cardiac output index (ml/min/kg)^¶^ | 290 (216-376) | 254 (217-296) | 0.056 | 274 (235-327) | 270 (201-295) | 0.444 |
|  | *Right diastolic function* | | | *Right diastolic function* | | |
| Tricuspid E/A ratio | 0.66 (0.63-0.73) | 0.72 (0.62-0.73) | 0.082 | 0.73 (0.68-0.82) | 0.75 (0.70-0.80) | 0.369 |
| Tricuspid inflow time fraction^$^ | 0.39 (0.37-0.43) | 0.38 (0.34-0.41) | 0.133 | 0.40 (0.35-0.43) | 0.35 (0.32-0.38) | **0.002** |
| Tricuspid A (atrial) time fraction^$^ | 0.23 (0.21-0.25) | 0.21 (0.20-0.23) | **0.046** | 0.23 (0.19-0.25) | 0.20 (0.18-0.22) | **0.049** |
| Data are presented as number (percentage) for qualitative variables or median (25th centile; 75th centile) for quantitative variables. p was adjusted for estimated fetal weight <10th centile.  A: atrioventricular peak velocitie at atrial contraction; E, atrioventricular peak velocitie at early diastole; *PTL: Preterm labor; US: Ultrasound*  * Right atrial (RA) or right ventricular (RV) area-to-heart area ratio = RA or RV area (cm2)/cardiac area (cm2)  ^†^ Ventricular sphericity index: ventricular longitudinal diameter (mm)/ transverse diameter (mm)  ^‡^ FAC: ((End-diastolic right ventricular area (cm2) – End-systolic right ventricular area (cm2)) / End-diastolic right ventricular area (cm2))*100  ^§^ Stroke volume: (Pulmonary area (cm2)* pulmonary VTI (cm/contraction))/EFW (Kg)  ^¶^ Right Cardiac output index: (Pulmonary stroke volume (ml/contraction) * heart rate (bpm)) / EFW (Kg)  ^$^ Diastolic times fraction: tricuspid inflow or A wave duration (ms) / total cycle duration (ms) | | | | | | |

| **TABLE S6: Left heart: fetal echocardiographic results at admission and at 4 weeks follow-up including only deliveries at term (> 37 weeks)** | | | | | | |
| --- | --- | --- | --- | --- | --- | --- |
| **Variables** | **First US (at admission)** | | | **Second US (1-month after admission)** | | |
|  | **Threatened** **PTL (n= 27)** | **Control (n=43)** | **p** | **Threatened** **PTL (n= 27)** | **Control (n=43)** | **p** |
|  | *Left cardiac morphometry* | | | *Left cardiac morphometry* | | |
| Left atrial area to heart ratio* | 0.17 (0.15-0.19) | 0.14 (0.13-0.18) | 0.059 | 0.15 (0.12-0.17) | 0.15 (0.13-0.17) | 0.460 |
| Left ventricular area to heart ratio* | 0.26 (0.24-0.31) | 0.26 (0.22-0.30) | 0.374 | 0.25 (0.22-0.28) | 0.24 (0.20-0.26) | 0.142 |
| Left ventricular sphericity index^†^ | 1.68 (1.45-1.92) | 1.79 (1.60-1.92) | 0.190 | 1.77 (1.57-1.83) | 1.86 (1.63-1.99) | 0.080 |
| Left ventricle free-wall thickness (mm) | 3.4 (2.4-3.9) | 2.8 (2.4-3.3) | 0.086 | 3.4 (3.0-3.9) | 3.6 (2.9-4.1) | 0.666 |
|  | *Left systolic function* | | | *Left systolic function* | | |
| MAPSE | 4.2 (3.8-5.0) | 4.2 (3.7-5.0) | 0.786 | 5.5 (4.1-6.1) | 4.9 (4.0-5.8) | 0.234 |
| Left ventricular shortening fraction (%)^‡^ | 0.42 (0.34-0.48) | 0.40 (0.34-0.48) | 0.919 | 0.439 (0.333-0.490) | 0.390 (0.311-0.451) | 0.180 |
| Left isovolumic contraction time index (ICT, %)^§^ | 8.6 (8.0-9.3) | 8.1 (6.8-9.4) | 0.112 | 8.3 (7.3-9.0) | 7.6 (6.7-9.5) | 0.364 |
| Left ejection time index (ET, %)^§^ | 39.8 (38.2-42.8) | 39.9 (38.5-42.0) | 0.730 | 38.2 (36.8-39.7) | 39.0 (37.3-40.9) | 0.334 |
| Aortic diameter (mm) | 4.6 (4.2-5.2) | 4.9 (4.4-5.4) | 0.599 | 5.6 (5.1-6.8) | 6.0 (4.9-6.8) | 0.941 |
| Peak systolic aortic velocity (cm/s) | 81.0 (75.7-90.8) | 78.7 (70.893.6) | 0.822 | 84.3 (73.4-95.0) | 84.9 (74.9-97.8) | 0.537 |
| Left stroke volume index (ml/kg)^¶^ | 1.28 (0.92-1.71) | 1.30 (1.06-1.60) | 0.969 | 1.10 (0.92-1.45) | 1.22 (0.95-1.68) | 0.997 |
| Left cardiac output index (ml/min/kg)^$^ | 183(139-236) | 187 (159-244) | 0.731 | 147 (132-204) | 169 (133-232) | 0.747 |
| Aortic isthmus PI | 2.15 (2.02-2.67) | 2.49 (2.20-2.66) | 0.180 | 2.50 (2.64-2.92) | 2.52 (2.26-2.70) | 0.069 |
|  | *Left diastolic function* | | | *Left diastolic function* | | |
| Mitral E/A ratio | 0.72 (0.66-0.75) | 0.73 (0.68-0.79) | 0.333 | 0.75 (0.69-0.82) | 0.72 (0.68-0.82) | 0.085 |
| Mitral inflow time fraction^+^ | 0.42 (0.39-0.44) | 0.43 (0.40-0.45) | 0.941 | 0.44 (0.40-0.46) | 0.42 (0.40-0.46) | 0.138 |
| Mitral A (atrial) time fraction^+^ | 0.22 (0.22-0.24) | 0.22 (0.20-0.24) | 0.381 | 0.23 (0.20-0.25) | 0.23 (0.21-0.24) | 0.172 |
| Left isovolumic relaxation time index (IRT, %)^§^ | 11.0 (10.3-11.8) | 11.9 (10.4-13.0) | 0.183 | 11.3 (10.7-13.4) | 12.0 (11.0-14.0) | 0.939 |
|  | *Left combined systolic and diastolic function* | | | *Left combined systolic and diastolic function* | | |
| Left MPI index^#^ | 0.50 (0.44-0.53) | 0.50 (0.44-0.56) | 0.716 | 0.54 (0.47-0.59) | 0.52 (0.48-0.58) | 0.569 |
| Data are presented as number (percentage) for qualitative variables or median (25th centile-75th centile) for quantitative variables. p was adjusted for estimated fetal weight <10th centile.  A: atrioventricular peak velocitie at atrial contraction; E, atrioventricular peak velocitie at early diastole; *PTL: Preterm labor; US: Ultrasound*  * Left atrial (LA) or left ventricular (LV) area-to-heart area ratio = LA or LV area (cm^2^)/cardiac area (cm^2^)^†^ Ventricular sphericity index: ventricular longitudinal diameter (mm)/ transverse diameter (mm)  ^‡^ Left ventricular shortening fraction (M-Mode): ((End-diastolic ventricular diameter (mm) - End-systolic ventricular diameter (mm)) / End-diastolic ventricular diameter (mm))*100  ^§^ IRT, ICT, ET indexes: (IRT, ICT or ET (ms) / total cycle duration (ms))*100; ^11^ MPI: (ICT (ms) + IRT (ms)) / ET (ms)  ^¶^ Stroke volume: (Aortic area (cm2)* aortic VTI (cm/contraction))/EFW (Kg)  ^$^ Left Cardiac output index: (Aortic stroke volume (ml/contraction) * heart rate (bpm)) / EFW (Kg)  ^+^ Diastolic times fraction: Mitral inflow or A wave duration (ms) / total cycle duration (ms)  ^#^ MPI: (ICT (ms) + IRT (ms)) / ET (ms) | | | | | | |

| **TABLE S7: Indications for amniocentesis in the Biobank samples.** | |
| --- | --- |
| Gestational age at amniocentesis (weeks+days) | Indication for amniocentesis |
| 26+3 | Congenital talipes equinovarus |
| 28+0 | Hyperechogenic fetal bowel |
| 28+3 | Moderate renal pyelectasis |
| 28+5 | Mild polyhydramnios + hyperechogenic fetal bowel |
| 28+6 | Mild renal pyelectasis |
| 29+3 | Hyperechogenic fetal bowel |
| 29+6 | Hyperechogenic fetal bowel |
| 31+4 | Hypospadias |
| 36+1 | Long bones at -1 to -2 standard deviations. |
| 36+3 | Long bones at -2 to -3 standard deviations. |
| 27+4 | 8-9mm right and left brain atrial diameters |
| 27+5 | 9-10mm right and left brain atrial diameters |
| 28+2 | Unilateral mild ventriculomegaly (10mm) |
| 29+2 | 9mm right brain atrial diameter |
| 29+6 | 8mm right brain atrial diameter |
| 31,2 | Mild delay in cortical maturation |
| 31+3 | 9 mm left brain atrial diameter |
| 32+6 | 9 mm right brain atrial diameter |
| 35+2 | 8 mm left brain atrial diameter |
| 37+4 | 3 mm arachnoid cyst |
| *All the amniocenteses were performed for extra-cardiac reasons. Pregnancies did not present risk factors for cardiac remodeling, and normal results were confirmed in the amniotic fluid analysis and healthy offspring were delivered in all cases.* | |

| **TABLE S8: Baseline characteristics Threatened PTL versus Biobank SF samples groups** | | | |
| --- | --- | --- | --- |
| Variables | Threatened PTL  (n= 32) | Biobank AF samples  (n=20) | p |
| GA at amniocentesis (weeks) | 29.4 (26.4;31.0) | 29.5 (28.3;32.0) | **0.033** |
| EFW at ultrasound (gr) | 1403 (1060;1766) | 1495 (1192;2012) | 0.075 |
| Neonatal male sex, n(%) | 22 (68.8%) | 12 (60.0%) | 0.519 |
| EFW centile | 63.5 (37.0;74.0) | 56.5 (32.0;77.5) | 0.690 |
| *Data are presented as number (percentage) for qualitative variables or median (25^th^ centile; 75^th^ centile) for quantitative variables.*  *PTL, preterm labor; EFW, estimated fetal weight; AF, GA, gestational age; AF, amniotic fluid.* | | | |

| **TABLE S9: Cardiac biomarkers in amniotic fluid at admission including only deliveries at term (>37 weeks)** | | | |
| --- | --- | --- | --- |
| Variables | Threatened PTL  (n= 27) | Biobank AF samples  (n=20) | p |
| Detectable NT-proBNP, n (%) | 8 (29.6%) | 0 | **0.006** |
| NT-proBNP (pg/ml)* | 1644.9 (466.3-3209) | - | NA |
| Troponin-I (pg/ml) | 1231.9 (827.2-1619.2) | 841.3 (671.7-960.0) | **0.034** |
| Data are presented as number (percentage) for qualitative variables or median (25th centile-75th centile) for quantitative variables. p was adjusted for gestational age at amniocentesis.  *AF, amniotic fluid; NA, not applicable; NT-proBNP, N-terminal- pro-B-type natriuretic peptide; PTL, preterm labor.*  *Including only the sample levels of detectable NT-proBNP.s | | | |
